# Auf dem Weg zu einer besseren Versorgung und Forschung bei Seltenen Erkrankungen

**DOI:** 10.1007/s00103-022-03604-0

**Published:** 2022-11-04

**Authors:** Stefanie Weber, Helge Hebestreit, Holm Graessner

**Affiliations:** 1 Abteilung „Kodiersysteme und Register“, Bundesinstitut für Arzneimittel und Medizinprodukte, Kurt-Georg-Kiesinger-Allee 3, 53175 Bonn, Deutschland; 2 Zentrum für Seltene Erkrankungen – Referenzzentrum Nordbayern, Universitätsklinikum Würzburg, Josef-Schneider-Str. 2, 97080 Würzburg, Deutschland; 3 Zentrum für Seltene Erkrankungen und Institut für Medizinische Genetik und Angewandte Genomik, Universitätsklinikum Tübingen, Calwerstr. 7, 72076 Tübingen, Deutschland

Die Themen „Seltene Erkrankungen“ und „Digitalisierung“ sind jeweils sehr relevante Themen für die Gesundheitsversorgung in Deutschland. Probleme, aber auch Chancen und Nutzen wurden für beide Themenbereiche bereits in mehreren Heften des *Bundesgesundheitsblatts* thematisiert. In diesem Schwerpunktheft haben wir beide Themen integriert und möchten aufzeigen, welche Chancen die Digitalisierung insbesondere für Menschen mit Seltenen Erkrankungen mit sich bringt. Dabei soll betont werden, dass Betroffene und Betroffenenvertretungen in die dargestellten Entwicklungen und Projekte eng eingebunden waren und sind. Es kann aber auch nicht verschwiegen werden, dass eine gleichberechtigte Partizipation sowohl politisch als auch auf der Ebene der konkreten Projekte und Initiativen oftmals noch nicht erreicht wurde.

Grundsätzlich haben die Vereinten Nationen am 16.12.2021 während ihrer 76. Sitzung in einer Resolution^1^ die Bedeutung von Daten für Menschen mit Seltenen Erkrankungen betont und ihre Mitgliedsstaaten aufgefordert, gemeinsam an der Bereitstellung und dem Teilen von Daten zu arbeiten.

Die Bedeutung der Digitalisierung für Menschen mit Seltenen Erkrankungen umfasst vielfältige Aspekte. Das Nationale Aktionsbündnis für Menschen mit Seltenen Erkrankungen nimmt entsprechend an vielen Stellen Bezug auf die Thematik und hat vielfältige Maßnahmen in die Wege geleitet. Im ersten Artikel von *Wessel et al.* wird hierzu eine Übersicht gegeben.

Um Daten zusammenführen und teilen zu können, ist der erste Schritt eine syntaktische und semantische Standardisierung. Einige hierfür entwickelte Systeme werden in dem Beitrag von *Robinson und Graessner* zusammengestellt und erklärt. Durch die unterschiedlichen Anwendungsfälle ist eine zwar heterogene, aber für Seltene Erkrankungen spezifische Landschaft von Kodiersystemen entstanden, die oftmals für Forschungszwecke konzipiert wurden, inzwischen aber mehr und mehr in der Versorgung Anwendung finden.

Wie stellt sich dies in der Versorgungspraxis dar? Im Beitrag von *Martin et al.* werden die praktische Anwendung von Kodiersystemen und die bevorstehende Einführung der spezifischen Kodierung mit ORPHAcodes erklärt und an Erfahrungsberichten aus 3 Zentren für Seltene Erkrankungen veranschaulicht. Hieraus ergeben sich Anregungen für andere Einrichtungen, die sich nun mit der Einführung auseinandersetzen werden, aber auch Ausblicke für mögliche nächste Schritte.

In Deutschland, wie auch in vielen anderen Ländern, stellt die elektronische Patientenakte einen Hoffnungsträger für den Nutzen der Digitalisierung dar. Patientinnen und Patienten sollen Zugriff auf ihre Daten haben und selbstbestimmt und informiert ihre Behandlung erhalten. Hierzu sind bereits 2 Ansätze für Menschen mit Seltenen Erkrankungen in Forschungsprojekten verfolgt worden, die im Beitrag von *Rashid et al.* geschildert werden. Erfahrungen, positive Ergebnisse, aber auch Hürden, die dabei aufgedeckt wurden, liefern wichtige Hinweise, die bei dem Ausrollen der bundesweiten elektronischen Patientenakte genutzt werden können.

Im Rahmen der Medizininformatik-Initiative (MII), einem vom Bundesministerium für Bildung und Forschung (BMBF) finanzierten umfangreichen Forschungsprogramm zur Zusammenführung von Daten im Gesundheitswesen, wurde ein Schwerpunkt auf den Anwendungsfall der Seltenen Erkrankungen gelegt. Im Artikel von *Schepers et al.* werden die Herangehensweise und die identifizierten Lösungswege des MII-Anwendungsfalles für Seltene Erkrankungen (Collaboration on Rare Diseases – CORD) geschildert.

Welchen Nutzen die künstliche Intelligenz (KI) in der Diagnostik von Seltenen Erkrankungen erzielen kann, wird anschaulich im Beitrag von *Krawitz* dargestellt. Durch die Analyse großer Datenmengen und das Trainieren einer KI auf Abweichungen im Phänotyp kann der diagnostische Prozess unterstützt und damit deutlich beschleunigt werden.

Die Gesamtanzahl der Menschen mit Seltenen Erkrankungen ist sehr groß, sieht man sich aber einzelne Erkrankungen an, so ist die Anzahl der Betroffenen oft sehr klein. Um ihnen den Zugang zu der bestmöglichen Versorgung bieten zu können und das aktuellste Wissen über die jeweilige Erkrankung zu bündeln, ist die grenzüberschreitende Zusammenarbeit von Expertinnen und Experten notwendig. Durch die Initiative zur Bildung von Europäischen Referenznetzwerken (ERN), einem Netzwerk von Expertenzentren, werden Diagnostik, Therapie und Forschung europaweit vernetzt. Im Beitrag von *Graessner et al.* werden die Initiative und die im Digitalisierungs- und Datenkontext erreichten Verbesserungen in der Versorgung geschildert sowie ein Ausblick auf die nächsten nötigen Schritte gegeben.

Im letzten Beitrag wird schließlich das Thema der „Orphan Drugs“, der Arzneimittel für Menschen mit Seltenen Erkrankungen, aus verschiedenen Perspektiven betrachtet und die Rolle der Daten im Zulassungs- und Nutzenbewertungsprozess analysiert. Auch in diesem Beitrag von *Naumann-Winter et al.* spielt die Standardisierung der Daten eine entscheidende Rolle.

Letztendlich zieht sich durch alle Beiträge im Heft ein roter Faden. Die Digitalisierung bietet für Menschen mit Seltenen Erkrankungen eine Chance, Diagnostik und Therapie und damit auch die gesamte Versorgung zu verbessern. Digitalisierung darf kein Selbstzweck sein, sondern muss immer auch mit einem Nutzen für die Patientinnen und Patienten verbunden sein. Konkret sind dies: schnellere Diagnosestellung, verbesserter Zugang zu den nötigen Informationen für die Behandlung, effektivere Therapie, bessere Forschungsmöglichkeiten bzw. Entlastung des Gesundheitspersonals und damit mehr Zeit für Patientinnen und Patienten. In den letzten Jahren wurde bereits viel erreicht und Menschen mit Seltenen Erkrankungen können im besonderen Maße von der Digitalisierung profitieren.

Basierend auf dem Erreichen sind allerdings weitere Schritte notwendig, um das Potenzial der Digitalisierung insbesondere für die Versorgung noch besser ausschöpfen zu können. Zukünftige relevante Entwicklungen können z. B. folgende sein: die Kodierung der Diagnose einer Seltenen Erkrankung mittels ORPHAcode auch im ambulanten Bereich, die Nutzung der Human Phenotype Ontology (HPO-)Terms zur standardisierten Beschreibung der Symptomatik, die routinemäßige Dokumentation des europäischen Kerndatensatzes für Seltene Erkrankungen an den Zentren für Seltene Erkrankungen und die (Weiter‑)Entwicklung einer elektronischen Patientenakte zum besseren Datenaustausch auch im klinischen Kontext.

Die Koordinatoren dieses Heftes hoffen, hiermit Ihr Interesse geweckt zu haben und Sie zum Nachdenken und zur Entwicklung weiterer Ideen anregen zu können.

## Footnotes

1 United Nations General Assembly. 76/132. Addressing the challenges of persons living with a rare disease and their families. Resolution adopted by the General Assembly on 16 December 2021. https://undocs.org/en/A/RES/76/132; Zugriff am 16.09.2022.

